# The effect of footwear on mechanical behaviour of the human ankle plantar-flexors in forefoot runners

**DOI:** 10.1371/journal.pone.0274806

**Published:** 2022-09-19

**Authors:** Jason Bonacci, Wayne Spratford, Claire Kenneally-Dabrowski, Danielle Trowell, Adrian Lai

**Affiliations:** 1 Centre for Sports Research, School of Exercise and Nutrition Sciences, Deakin University, Geelong, Australia; 2 Movement Science, Australian Institute of Sport, Canberra, Australia; 3 Discipline of Sport and Exercise Science, Faculty of Health, University of Canberra, Canberra, Australia; 4 University of Canberra Research Institute for Sport and Exercise (UCRISE), University of Canberra, Australia; 5 Lululemon Athletica, Vancouver, Canada; The Wingate College of Physical Education and Sports Sciences at the Wingate Institute, IL, ISRAEL

## Abstract

**Purpose:**

To compare the ankle plantar-flexor muscle-tendon mechanical behaviour during barefoot and shod forefoot running.

**Methods:**

Thirteen highly trained forefoot runners performed five overground steady-state running trials (4.5 ± 0.5 m^.^s^-1^) while barefoot and shod. Three-dimensional kinematic and ground reaction force data were collected and used as inputs for musculoskeletal modelling. Muscle-tendon behaviour of the ankle plantar-flexors (soleus; medial gastrocnemius; and lateral gastrocnemius) were estimated across the stance phase and compared between barefoot and shod running using a two-way multivariate analysis of variance.

**Results:**

During barefoot running peak muscle-tendon unit (MTU) power generation was 16.5% (p = 0.01) higher compared to shod running. Total positive MTU work was 18.5% (p = 0.002) higher during barefoot running compared to shod running. The total sum of tendon elastic strain energy was 8% (p = 0.036) greater during barefoot compared to shod running, however the relative contribution of tendon and muscle fibres to muscle-tendon unit positive work was not different between conditions.

**Conclusion:**

Barefoot forefoot running demands greater muscle and tendon work than shod forefoot running, but the relative contribution of tendon strain energy to overall muscle-tendon unit work was not greater.

## Introduction

The human ankle plantar-flexors, the soleus (SOL), medial gastrocnemius (MG) and lateral gastrocnemius (LG), perform an important biomechanical function during running. Experimental and musculoskeletal modelling studies have demonstrated that the ankle plantar-flexors generate force up to 12 times body weight (BW) during running [1]; the greatest force of all the lower-limb muscle groups. They are also the dominant contributors to support and horizontal propulsion of the body’s centre of mass during running [2, 3]. The ankle plantar-flexors have relatively short muscle fibres that insert onto the calcaneus via a compliant Achilles tendon. This configuration favours the capacity to store elastic strain energy, which contributes a considerable amount of the mechanical work performed by the musculotendon units [4, 5]. Previous modelling studies have demonstrated that elastic strain energy stored in the Achilles tendon during steady-state running provides a greater contribution to muscle-tendon unit (MTU) propulsive work compared to the muscle fibres [6–8]. The contribution of tendon strain energy to overall MTU propulsive work increases as running speed advances from slow running towards maximum sprinting [8]. However, the effects of footwear and foot-strike type on the energetics of the muscle fibres and tendon in the ankle plantar-flexors during running remain largely unknown.

Sinclair et al. [9] utilised musculoskeletal modelling to examine the effect of footwear on ankle plantar-flexor muscle forces during running. They reported a 32% increase in MG muscle force during barefoot running compared to shod running, but no difference in LG or SOL muscle forces between conditions. Foot-strike pattern was not controlled in the study. For example, the ankle was more plantarflexed at contact in the barefoot running condition suggesting that participants switched from a rearfoot strike during the shod condition to a mid/forefoot strike during the barefoot condition. It is not possible to discern if the increase in force developed by the MG was due to differences in footwear or foot-strike type. Running barefoot and in minimalist shoes has been associated with greater peak internal ankle plantarflexion moments and plantar-flexor impulse during stance [10, 11]. These greater demands on the ankle plantar-flexors are due to a lack of shoe cushioning, which increases the muscular effort required to attenuate ground impact forces while potentially increasing the metabolic cost of running [12, 13]. However, running barefoot and in a minimalist shoe are more economical than running in a cushioned shoe, even when shoe mass is accounted for [11, 14]. If barefoot running is more economical but has greater ankle plantar-flexor demands, this discrepancy may suggest that the ankle plantar-flexors utilise greater Achilles tendon elastic strain energy during barefoot compared to shod running.

Perl et al. [11] postulated that the elevated heel during shod running and greater lower extremity elastic energy storage may explain the moderate metabolic benefits of barefoot or minimally shod running. The authors indirectly measured Achilles tendon strain using the overall length change of the entire triceps surae MTU complex. This method does not distinguish between the length changes of the muscle fibre and tendon components, which previous in-vivo studies of the ankle plantar-flexors during running have shown are decoupled from that of the MTU [15, 16]. As a result, it is not possible to differentiate the work done by the MTU in the ankle plantar-flexors into the elastic strain energy stored in the tendon and the work done by the muscle fibres. Therefore, the aim of the study was to investigate the effect of footwear on the mechanical behaviour of the ankle plantar-flexor muscle fibre and tendon components during running. Specifically, we used experimental kinematic and kinetic data in conjunction with musculoskeletal modelling to compute the mechanical power and work performed by the MTU, muscle fibres and tendon in the SOL, MG and LG during barefoot and shod running. As the purpose of this study was to examine the effect of footwear rather than foot strike on plantar-flexor MTU behaviour, only habitual forefoot runners were recruited. This is because rearfoot strikers often switch to a forefoot strike when running barefoot [17], which could confound the results. We hypothesised that in comparison to shod running, barefoot running would result in: (i) a greater amount of tendon elastic strain energy in the ankle plantar-flexors and; (ii) a greater relative contribution of tendon elastic strain energy contribution to MTU positive work compared to muscle fibre work.

## Methods

### Participants

Thirteen highly trained distance runners (8 males and 5 females; mean ± SD; age, 29.9 ± 5.9 years; height, 176.7 ± 7.5 cm; body mass, 64.9 ± 8.8 kg) were recruited for the study. Based on a priori sample size calculation, 13 participants would be sufficient to generate an effect size of 0.93 at a power of 80% and α of < 0.05 [9]. All participants were training for competition (training history: 14.3 ± 1.9 km per session, 7.4 ± 1.9 sessions per week, 109.6 ± 27.9 km per week) with the average personal best 10 km time in the previous year of 33.7 ± 3.7 minutes. All participants self-reported as a forefoot striker and this was confirmed during data collection. No participants were suffering from any pre-existing musculoskeletal injury that might affect their ability to participant in the study. Written informed consent was obtained from all participants and ethical approval was attained from Deakin University and Australian Institute of Sport human research ethics committees.

### Experimental data collection

Running trials were conducted on a 110 m indoor synthetic track in the Biomechanics Laboratory at the Australian Institute of Sport, Canberra. Three-dimensional kinematic data were collected using a 22-camera motion analysis system (VICON, Oxford Metrics Ltd, Oxford, UK) sampling at 250 Hz. The calibrated capture volume was approximately 20 m in length and situated ~60 m along the track, which allowed sufficient distance for participants to accelerate, hold a steady-state speed through the capture volume and then safely decelerate to rest. Retro-reflective markers (14 mm diameter) were placed at predefined locations on the pelvis and lower limbs [10]. Individual markers were placed on the left and right anterior superior iliac spines and posterior superior iliac spines. The thigh segment was defined by a three-marker cluster affixed laterally and aligned with the head of the femur and lateral femoral condyle. The lower leg was defined by a three-marker cluster aligned with the lateral femoral condyle and lateral malleolus. Markers placed on the superoposterior aspect of the calcaneus, and first and fifth metatarsals defined the foot. In the shod condition, these markers were placed on the shoe. Individual retroflective markers were also placed on the medial and lateral femoral condyles and medial and lateral malleoli to define the knee and ankle joint centres, respectively. Ground reaction force (GRF) data were collected using eight in-ground force plates (Kistler Instrument Corp., Dimensions: 900 x 600 mm, Amherst, New York, USA) sampling at 1500 Hz. The force plates were embedded into the synthetic running track directly adjacent to each other spanning a total length of 7.2 m. Marker trajectories and GRF data were filtered using fourth-order, low-pass Butterworth filters with the same cut-off frequency of 20 Hz [18].

The data collection protocol involved two experimental conditions: shod (lightweight racing flat, NIKE LunaRacer 2) and barefoot. The racing flat had a low heel-forefoot offset (6 mm) and mean mass of 184.2 ± 19.4 g. All participants were required to complete a 10-day familiarisation period prior to testing to get accustomed to the barefoot and shod conditions. The average distances completed by all participants during the familiarisation period in the barefoot and shod conditions were 4.3 ± 3.2 km and 20.7 ± 11.5 km in 2.6 ± 0.6 and 3 ± 0.6 sessions, respectively. The volume of barefoot familiarisation was lower than shod running to minimise likelihood of acute overload during this unfamiliar condition [19, 20]. All participants habitually wore a standard cushioned running shoe for most of their training volume.

Participants performed a standardised warm-up prior to data collection that involved five overground running trials within the capture volume. Participants then performed a static calibration trial and five overground steady-state running trials in each of the two randomly ordered conditions. The desired steady-state running speed for each participant was set at 90% of participant’s best 10 km time in the previous year. The mean desired steady-state running speed for all participants was 4.5 ± 0.5 m^.^s^-1^. Average steady-state speed for each trial were obtained using timing gates (Speedlight Telemetry Timing, Swift Performance Equipment, Walcol, QLD, Australia) placed at the start and end of the calibrated capture volume. Trials were accepted if the average speed was within ±5% of the desired speed. Steady state running was confirmed post testing via examination of the net horizontal force impulse (S1 Dataset). Forefoot strike was defined as a foot strike in which the point of first contact of the foot with the ground was the forefoot or the front half of the shoe sole. Two classification techniques were used to confirm that all participants had forefoot strike patterns. The first classification technique used the presence of an initial ankle plantar-flexion angle in kinematic data and absence of impact peak in the vertical GRF profile [17] while the second classification technique used the markers placed on the heel and the toe to define foot strike patterns [21]. The difference in vertical position of the heel and first metatarsal markers was calculated during both the static trial and at initial contact during running. The vertical difference between markers during the static trial was then subtracted from the difference at initial contact. Participants were classified as forefoot strikers if the final value was 40 mm or less [21]. Both classification techniques verified that all participants had forefoot strike patterns in both the barefoot and shod conditions. All participants completed both experimental conditions.

### Musculoskeletal model

The skeletal system was modelled as a 12 segment, 31 degree of freedom (DOF) mechanical linkage system, similar to that described by Hamner at al. [22]. The lower limb joints were modelled as follows: the pelvis was free to translate and rotate in space (6 DOF), the hip was a ball-and-socket joint (3 DOF), the knee was a hinge joint (1 DOF), and the ankle-subtalar complex was a universal joint (2 DOF) comprised of two non-intersection hinge joints. While the model contained the metatarsophalangeal joint, this was locked for all simulations and the mid and fore- foot acted as a rigid segment. The model was actuated by 96 MTU actuators. Each MTU was modelled as a Hill-type muscle consisting of in-parallel active and passive muscle fibre elements attached in-series with a series elastic element. Hereafter, the series elastic element will be termed tendon as a result of the significant influence of the free tendon on series elasticity compared with other elastic connective tissue (e.g. aponeurosis). Maximum shortening velocity was set to 15 optimal fibre lengths per second to be consistent with previous modelling studies investigating running [23, 24]. The maximum isometric force of all muscles was increased three-fold, as required for successful simulations of highly dynamic movement such as gait [25, 26].

The SOL, MG and LG were assumed to be three separate MTUs with three independent tendon elements representing the Achilles tendon. The tendon strains at maximum isometric force generation were informed by previously reported data. Experimental data from three rearfoot runners [16] were used to evaluate the effect of modifying plantar-flexor tendon strains on model-based estimates of SOL and MG muscle fibre lengths. Simulated fibre lengths were compared to that measured in-vivo using ultrasound during rearfoot running [6, 16, 27] across a range of tendon strains from 3.3%-10%. A tendon strain of 5% for the SOL, MG and LG gave the most comparable model-based muscle fibre length changes in the SOL and MG during stance phase. Tendon strains at maximum isometric contraction for the ankle plantar-flexors were consistent with reported in-vivo measurements of the Achilles tendon of 4.9 ± 1% [28] and 5.1 ± 1.1% [29]. Furthermore, simulated tendon strains during running were within the range of tendon strains reported using dynamic ultrasound measurements of the SOL and MG at equivalent running speeds [15, 16]. These two consistencies support our decision to increase the tendon strain for the ankle plantar-flexors at maximum isometric force generation. Tendon compliance in other MTUs remained unchanged at 3.3% of maximum isometric force generation [30].

### Computational simulations

Muscle modelling simulations were performed using OpenSim^TM^ [31]. Subject-specific musculoskeletal models were attained by scaling a generic model to the participant’s height and body mass. Individual musculoskeletal models were created for each participant and experimental condition (i.e. barefoot and shod). The same generic model (with identical marker placement) was used during scaling for each condition. A set of joint angles for each time instant was calculated using an inverse kinematic analysis where the sum of the squares of the differences between experimental markers trajectories and virtual markers in the model was minimised [32].

An inverse dynamics analysis in conjunction with a computed muscle control algorithm (CMC) were used to predict muscle forces and activations [33, 34]. A standard inverse dynamic approach was used to compute net joint torques generated about the torso, hip, knee and ankle joints. Residual reduction analysis (RRA) was used to reduce dynamic inconsistencies between joint kinematics and the measured GRF. The errors in the residual forces and dynamically-consistent kinematics were within the recommended bounds of the analysis [35]. Muscle forces and activations were computed using CMC in accordance with the physiological force-length and force-velocity properties of muscle fibre and tendon, as well as the geometric and dynamic constraints of the system. CMC solved the muscle redundancy problem by predicting a set of muscle excitations that drove a model forward in time (simulation time window of 0.01 s) such that the sum of squared muscle activations was minimised and the kinematics of the model tracked the dynamically consistent joint kinematics obtained from RRA. Muscle excitations were bounded between 0 (no muscle activation) and 1 (full muscle activation) with activation and deactivation time constants of 10 and 30 ms, respectively [30].

The mechanical power developed by the MTU, muscle fibre and tendon elements were calculated by multiplying MTU, muscle fibre and tendon force by their corresponding contraction velocity at each time instant. Negative and positive power represented power absorption and generation, respectively. The positive work done by the MTU, muscle fibre and tendon was found by integrating the MTU, muscle fibre and tendon power curves over the duration of the stance phase where power was generated. All participants were forefoot strikers, thus the tendon and MTU lengthened and performed negative work during early stance followed by a period of positive work during mid- to late stance. The recovery of tendon elastic strain energy was represented by the positive work done by the tendon after the tendon performed negative work and after the MTU started generating positive power.

In this study, we were specifically interested in the percentage contributions of positive muscle fibre work and tendon elastic strain energy to the positive work done by the MTU (i.e., propulsion energy). The calculation of these contributions are detailed in a previous paper [8]. Briefly, the contributions were calculated using the following equation,

$$\%\mathrm{MTU}\;\mathrm{contribution}=\frac{W}{W_{MTU}}\times 100$$

where *W*_*MTU*_ is the positive work done by the MTU and *W* is the area under the *W*_*MTU*_ curve attributable to positive muscle fibre work or tendon elastic strain energy.

### Data analysis

Data for each participant were averaged over five stance phases for each experimental condition; time normalised to 0–100% of the stride cycle and used to calculate group mean ± SD values. Muscle force was normalised by the participant’s body weight while mechanical power and work were normalised by body mass (kg). Stride parameters of stance duration and stride length were calculated, along with the relative heel-toe marker position to classify footstrike position. The outcome variables included SOL, MG and LG peak muscle force (BW), peak MTU positive power (W^.^kg^-1^), total MTU positive work (J^.^kg^-1^) and the percentage contributions of positive muscle fibre work and tendon elastic strain energy to the positive work done by the MTU. The total sum of tendon elastic strain energy stored in all three ankle plantar-flexors was also calculated. Data are presented as mean ± SD. The Shapiro-Wilk test was conducted to determine if any data violated the assumption of normality. Stance duration, stride length, footstrike position and total sum of tendon elastic strain energy were compared between shod and barefoot conditions using a two-tailed paired sample t-test with α set at < 0.05. A two-way multivariate analysis of variance (MANOVA) was used to identify the effect of footwear and muscle on ankle plantar-flexor peak muscle forces, peak MTU power generation, total positive MTU work, and contribution of tendon and muscle fibre to positive MTU work. Where a main effect was found, post-hoc tests with Bonferroni correction were used to test for differences between means. Standardised mean differences (SMD) were calculated to express the magnitude of difference between conditions and interpreted according to the following criteria: calculated SMD of 0.2–0.49, small change; SMD of 0.5–0.79, moderate change; and SMD ≥ 0.8, large change [36]. All statistical analysis was conducted using the Statistical Package for the Social Sciences v27 (IBM Statistics, Chicago, USA).

## Results

**Fig 1:**
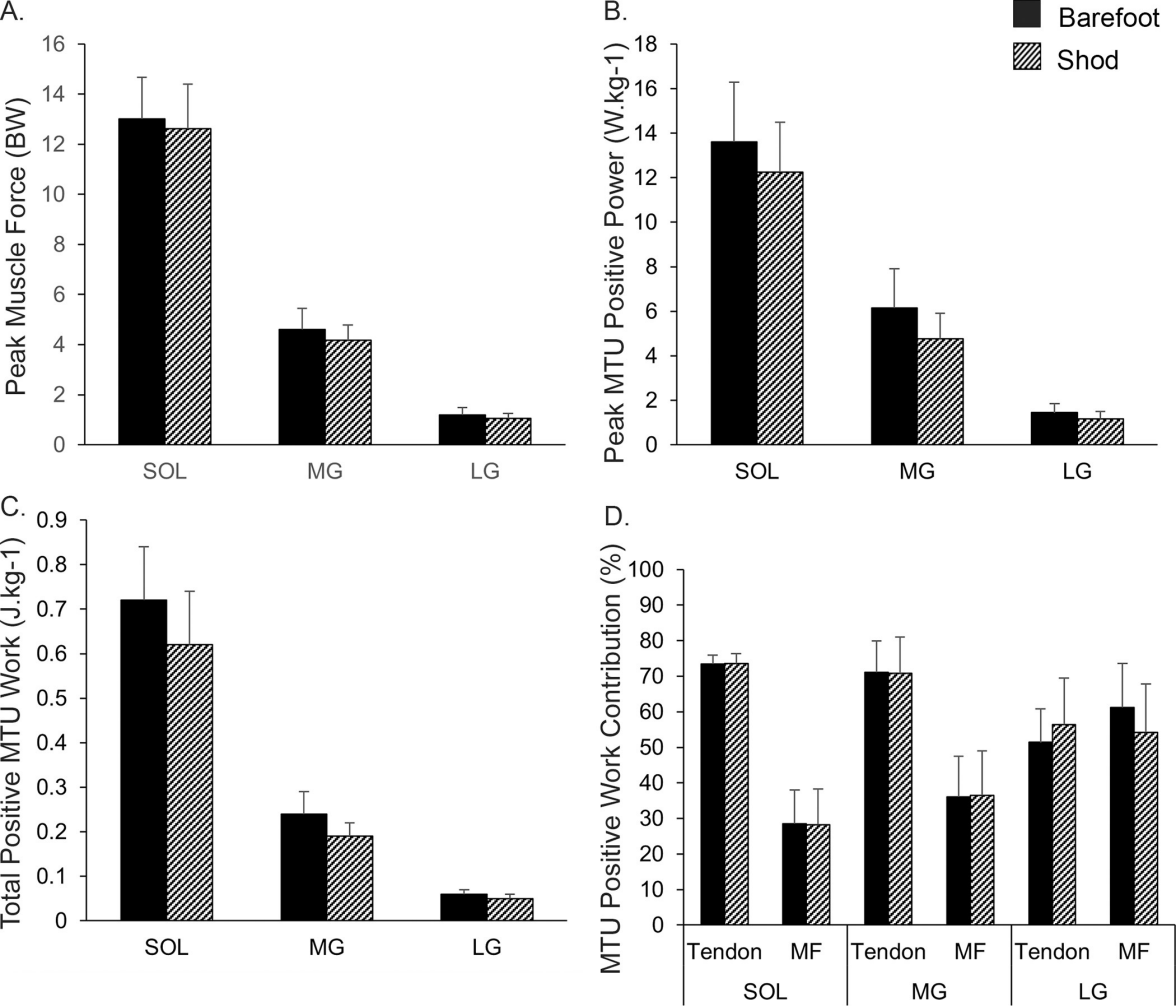
(A) Peak MTU muscle force; (B) peak MTU power generation; (C) total positive MTU work; (D) relative tendon and muscle fibre contributions to positive work (%) during the stance phase of barefoot and shod running.

Stance duration and footstrike position were not different between barefoot and shod running, though there was a small decrease in stride length during barefoot compared to shod running (Table 1). Peak muscle force, peak MTU power generation, total positive MTU work and relative contribution of tendon and muscle fibre to MTU positive work are plotted in Fig 1. Plantar-flexor muscle fibre, tendon and MTU length curves across the stance phase are presented in S1 Appendix.

**Table 1:** Group mean ± SD values and the difference between footwear conditions for stride parameters and footstrike position.

	Barefoot	Shod	Mean difference [95% CI]	p-value	SMD
Stance duration (s)	0.2 ± 0.01	0.2 ± 0.01	0.00 [-0.00, 0.00]	0.391	0.098
Stride length (m)	3.0 ± 0.3	3.1 ± 0.3	-0.1 [-0.11, -0.07]	0.001*	0.29^†^
Footstrike position (mm)^#^	34.4 ± 3.6	33.2 ± 3.6	1.2 [-0.3, 2.8]	0.099	0.34

^#^A lower relative value indicates more ankle plantarflexion at initial contact (forefoot strike < 40 mm).

*Significant difference between barefoot and shod conditions. ^†^ Small change

The total sum of tendon elastic strain energy was 8.3% (1.2 to 15.5%, SMD = 1.17, p = 0.036) higher during barefoot (0.9 [0.1] J^.^kg^-1^) compared to shod (0.8 [0.1] J^.^kg^-1^) running. The two-way MANOVA revealed a main effect for footwear (p = 0.032) but no footwear by muscle interaction effects (p = 0.669). Univariate analysis demonstrated a significant difference in peak MTU power generation (p = 0.01) and total positive MTU work (p = 0.002) for footwear conditions. Compared to shod running, peak MTU power generation was 16.5% (4 to 28.9%, SMD = 0.7) higher when running barefoot. Total positive MTU work was 18.5% (7 to 30%, SMD = 0.92) higher during barefoot compared to shod running. There was no effect of footwear on peak muscle force (p = 0.21) or tendon and muscle fibre contribution to positive MTU work (p = 0.46 & 0.37, respectively).

## Discussion

The aim of this study was to investigate the mechanical behaviour of the plantar-flexor muscle fibre and tendon components during barefoot and shod forefoot running. Tendon elastic strain energy was significantly greater (8.3%, SMD = 1.2) during barefoot running compared to shod running, confirming our first hypothesis. Achilles tendon elastic strain energy contributed the majority of MTU positive work during both shod and barefoot running. This is consistent with previous studies of shod running [6–8], although estimates of SOL relative tendon contribution were approximately 10% greater than previously reported at comparative speeds [7, 8]. This discrepancy may be due to methodological differences, as previous studies did not control footstrike pattern and modelled the LG and MG as a single MTU [7, 8]. The relative contribution of the tendon to plantar-flexor MTU positive work was not different between barefoot and shod conditions. This indicates that both tendon and muscle fibre work are increased when running barefoot. This finding refutes our second hypothesis.

The sum of muscle fibre and tendon contributions to MTU positive work exceeded 100% for all plantar-flexors during both barefoot and shod running. However, this is expected and can be explained by the transfer of energy from the muscle fibre to the tendon. During early stance, muscle fibres of the plantar flexors shorten and do positive work, while the tendon and MTU lengthen and do negative work [16]. This behaviour is common in MTUs where the tendon is compliant [30], such as the plantar-flexors. Positive work done by the muscle fibres during early stance is transmitted to the tendon as it stretches. This results in energy stored in the tendon, which is later returned as the tendon shortens during propulsion, in late stance. Because of this additional energy returned via the tendon, the sum of tendon and muscle fibre positive work exceeds 100% of MTU positive work.

Peak muscle force was not significantly greater during barefoot compared to shod forefoot running. In contrast, Sinclair et al. [9] found large increases in MG (32%) peak forces during barefoot compared to shod running. This disparity may be due to differences in footstrike patterns between studies. In the current study, all participants used a forefoot strike during shod and barefoot running. In comparison, a switch from rear to mid/forefoot was noted between shod and barefoot running in the previous study. The observed change in footstrike pattern has previously been reported in habitual rearfoot shod runners when transitioning to barefoot running [17]. The large increases observed by Sinclair et al. [9] are likely due to a lack of cushioning combined with a change in footstrike pattern during barefoot running. An absence of shoe cushioning during barefoot running increases the ankle plantarflexion moment during stance due to an increased muscular effort to absorb impact forces [10, 11]. The increase in MG muscle forces and Achilles tendon strain energy during barefoot forefoot running must be carefully considered as a rapid transition out of footwear may overload this complex.

Peak MTU power generation and MTU total positive work increased when running barefoot compared to shod running. Previous studies have reported that greater positive work is required at the ankle joint as shoe cushioning decreases or is removed when running barefoot [10, 37]. This is likely caused by greater propulsive forces when running barefoot [38]. As the plantar-flexors are the primary contributors to ankle joint torque and positive work generation during propulsion, it is expected that greater work is required of the plantar-flexor MTUs when running barefoot. As previously noted, this increased MTU positive work was a result of increases in both tendon elastic strain energy and muscle fibre work. There is some evidence that barefoot running has economical benefits [11, 14]. However, the mechanism behind this is unclear. Perl et al. [11] postulated that greater elastic energy storage and release could explain greater economy when running barefoot. While the current study showed an increase in tendon elastic strain energy during barefoot running, the relative contribution of the tendon to MTU positive work was not greater. Thus, barefoot running was not associated with greater tendon contribution to overall MTU positive work. The muscle fibre contributions to overall MTU positive work were similar in both conditions. The overall increase in MTU work and similar tendon and muscle fibre contributions to MTU positive work are unlikely to contribute to an economical advantage during barefoot running.

We found increased Achilles tendon elastic strain energy while running barefoot compared to shod. While increased tendon elastic strain energy may be beneficial, we must also consider how tendon compliance affects the operating range of muscle fibres on the force-length curve. Increased tendon elastic-energy storage and recovery may result in muscle fibres operating almost isometrically, which is most beneficial if near the optimal operating length on the force-length curve [39]. However, a trade-off may occur if tendon compliance results in muscle fibres operating at lengths which are further from optimal. This was observed by Uchida et al. [40], whereby increased tendon compliance of the MG resulted in muscle fibres remaining shorter during running, and operating far from their optimal lengths. As a result, greater activation was required to produce the necessary plantarflexion moment and the metabolic power requirement of running was increased. A similar trend has been observed when examining plantar-flexor muscle-tendon behaviour at varying running speeds [8]. As speed increases, tendon contribution to MTU positive work increases; however, muscle fibres operate at progressively unfavourable regions of the force-length curve. In the current study, muscle fibre lengths and shortening were similar between barefoot and shod conditions (S1 Appendix). Therefore, in both conditions the plantar-flexor muscle fibres were at similar operating lengths. Both tendon contributions to work, and the resulting effects on muscle fibre length operating range must be considered when understanding the effects of fibre and tendon behaviour on running economy.

Previous studies show that increased ankle work when running barefoot or in minimalist shoes is associated with a decrease in mechanical work at the knee [10, 37]. Fuller et al. [37] suggested the shift in mechanical work towards the ankle may allow greater elastic-energy storage and recovery in the Achilles tendon, and therefore improve mechanical efficiency. The plantar-flexors have short fibres and a long, compliant tendon while the MTUs supporting the knee are generally larger with relatively shorter tendons [41]. Therefore, it could be more economical to rely on the plantar-flexors to produce power during stance [37]. A more holistic view of lower-limb positive work production may suggest that increased reliance on the ankle will result in increased elastic-energy storage and recovery, as this is better facilitated by the plantar-flexors. Further studies which examine the muscle and tendon mechanical behaviour of the MTUs supporting both the ankle and knee during forefoot shod and barefoot running are required to further explore this theory.

We observed an increase in total Achilles tendon elastic strain energy when running barefoot. Footwear also limits the compression and recoil of the elastic elements supporting the longitudinal arch of the foot [42]. The current study used a rigid mid and fore- foot model that does not consider the compression and recoil of the foot longitudinal arch. This is a limitation of the current study, as a simplified foot model may result in overestimation of predicted ankle joint power and therefore plantar-flexor MTUs power [43]. It is possible that this could influence differences in MTU power of the plantar-flexors between barefoot and shod running. Further, the small decrease (0.1 m) in stride length during barefoot running may explain the changes in plantar-flexor MTU behaviour between barefoot and shod running.

Predictions of plantar-flexor energetics are sensitive to the musculoskeletal model input parameters. The model and its underlying parameters were scaled to each subject’s height and mass; however subject-specific bone geometries and musculotendon measures were not utilised. In particular, Achilles tendon compliance can vary greatly in humans [44] and influences the magnitude of muscle fibre work and tendon elastic strain energy [39]. This is important to acknowledge within the context of this study. However, a robust method was used to determine the most appropriate tendon compliance, and this resulted in fibre [6, 16, 27] and tendon [15, 16] strains that were consistent with ultrasound measurements during steady-state running. The Achilles tendon was also represented within the model as three separate tendons, rather than one common tendon. While the exact effects of this design on predictions of plantar-flexor energetics is unclear, non-uniform deformation of tendinous regions arising from each plantar-flexor have been reported [45] and may support this modelling approach. We only examined the acute effects of barefoot forefoot running on plantar-flexor energetics. Long-term use of minimalist shoes and running barefoot can result in adaptations to the mechanical properties of the tendon. Runners with four years of running in minimalist shoes displayed greater Achilles tendon stiffness compared to traditionally shod runners [46] and the Achilles tendon adapts to increased loading when exposed to minimalist shoe running by increasing stiffness [47]. This suggests that greater Achilles tendon stiffness might also be seen in habitual barefoot runners. The mechanical properties of the tendon were the same during the barefoot and shod simulations. This study does not account for adaptations to the mechanical properties of the tendon that may occur with prolonged barefoot running, which may influence predicted plantar-flexor energetics. Finally, we used the same performance criterion to estimate muscle-tendon parameters during barefoot and shod running. It is possible that participants utilised a different cost function to minimise muscle excitations than the one used in this study; however, there is currently no evidence to support this.

In conclusion, running barefoot with a forefoot strike increased plantar-flexor positive work and power generation and Achilles tendon elastic strain energy when compared to shod forefoot running. The relative contribution of tendon to plantar-flexor MTU positive work remained similar between shod and barefoot forefoot running. These results indicate that barefoot forefoot running does not preferentially favour elastic energy over muscle fibre work more than shod running. Those who adopt barefoot forefoot running should be aware of the greater demand on the plantar-flexors and Achilles tendon compared to shod forefoot running. Future studies should consider how mechanical adaptations of the Achilles tendon in habitual barefoot runners may influence plantar-flexor energetics.

## Supporting information

S1 Appendix Plantar-flexor muscle fibre, tendon and musculotendon normalised lengths. (TIF)

S1 Dataset Individual data for the plantar-flexor muscle, tendon and musculotendon units during barefoot and shod forefoot running. (XLSX)
