# Supplementary figures and images for: The effect of footwear on mechanical behaviour of the human ankle plantar-flexors in forefoot runners
Source: PLoS One. 2022 Sep 19;17(9):e0274806. doi: 10.1371/journal.pone.0274806 (PMC9484631; doi:10.1371/journal.pone.0274806)

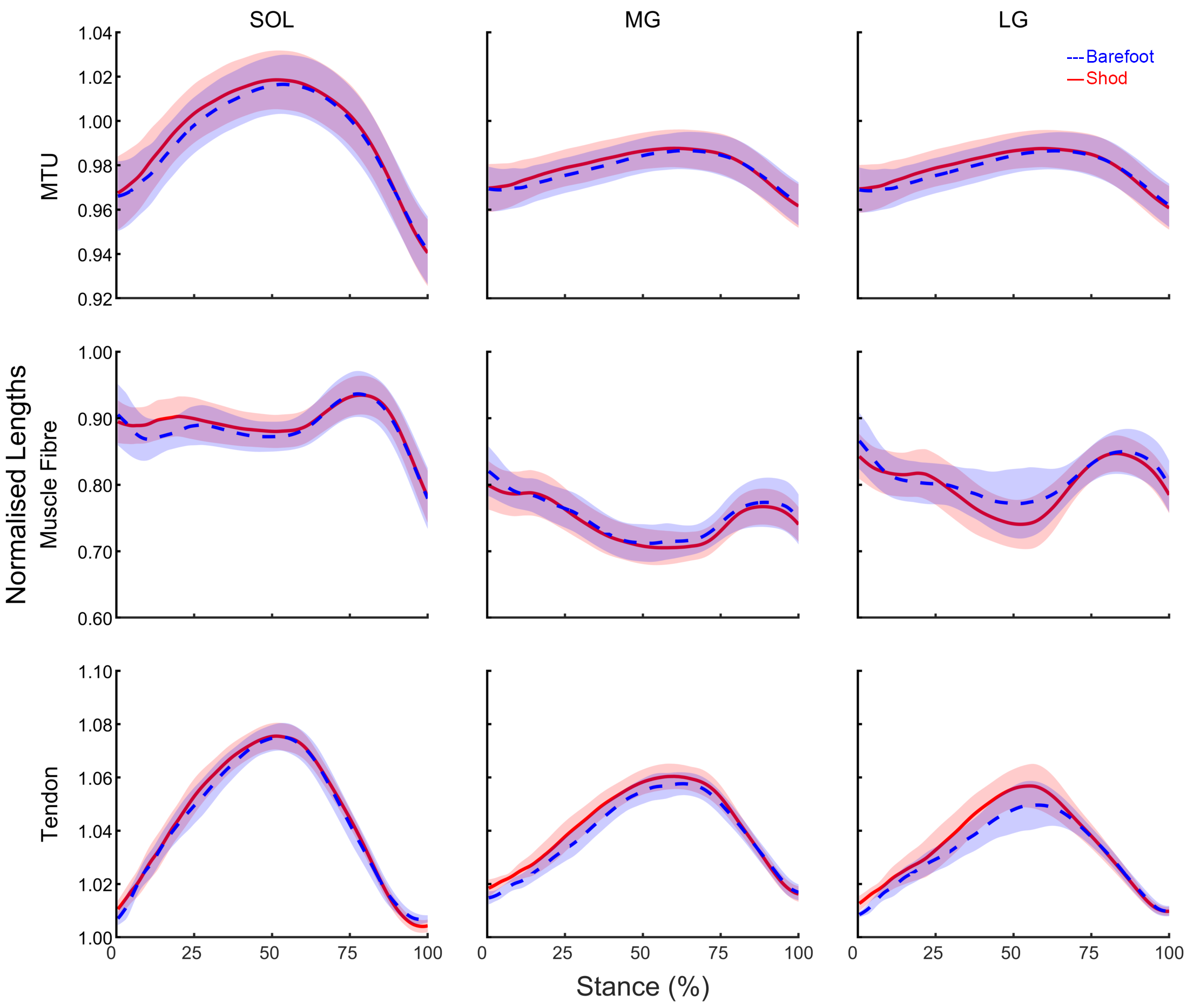

Supplement: S1 Appendix — (TIF) [file pone.0274806.s001.tif]
